# Characterization of Dof Transcription Factors and Their Responses to Osmotic Stress in Poplar (*Populus trichocarpa*)

**DOI:** 10.1371/journal.pone.0170210

**Published:** 2017-01-17

**Authors:** Han Wang, Shicheng Zhao, Yuchi Gao, Jingli Yang

**Affiliations:** 1 State Key Laboratory of Tree Genetics and Breeding, Northeast Forestry University, Harbin, Heilongjiang, China; 2 School of Pharmacy, Harbin University of Commerce, Harbin, China; 3 Annoroad Gene Technology Co., Ltd, Beijing, China; Nanjing Agricultural University, CHINA

## Abstract

The DNA-binding One Zinc Finger (*Dof*) genes are ubiquitous in many plant species and are especial transcription regulators that participate in plant growth, development and various procedures, including biotic and abiotic stress reactions. In this study, we identified 41 *PtrDof* members from *Populus trichocarpa* genomes and classified them into four groups. The conserved motifs and gene structures of some *PtrDof* genes belonging to the same subgroup were almost the same. The 41 *PtrDof* genes were dispersed on 18 of the 19 *Populus* chromosomes. Many key stress- or phytohormone-related *cis*-elements were discovered in the *PtrDof* gene promoter regions. Consequently, we undertook expression profiling of the *PtrDof* genes in leaves and roots in response to osmotic stress and abscisic acid. A total of seven genes (*PtrDof14*, *16*, *25*, *27*, *28*, *37* and *39*) in the *Populus Dof* gene family were consistently upregulated at point in all time in the leaves and roots under osmotic and abscisic acid (ABA) stress. We observed that 12 *PtrDof* genes could be targeted by 15 miRNAs. Moreover, we mapped the cleavage site in *PtrDof30* using the 5’RLM-RACE. The results showed that *PtrDofs* may have a role in resistance to abiotic stress in *Populus trichocarpa*.

## Introduction

Respond to specific signals by gene expression to resist damage and to survive in complicated environments, including specific metabolic and physiological pathways. Abiotic stresses, such as drought treatment and irregular temperature, strongly impact the development and growth of plants and have adverse effects on production and quality. In general, regulation of particular gene transcription has a critical impact on many biological and evolutionary processes in plants, for instance, stress and hormone responses. It is evident that transcription factors (TFs) are essential components that regulate transcriptional rates of their target genes by binding to *cis*-regulatory elements in the promoters.

The DNA-binding One Zinc Finger (Dof) transcription factor family contains a zinc finger domain, and plays critical roles as plant-specific transcriptional regulators in vital processes and functions in higher plants, such as stress response, photosynthetic carbon assimilation, dormancy and seed germination. Dof transcription factors typically contains 200–400 amino acids, among them, there was 52 amino acid with high level of conservation at the N-terminal, which has been considered as a DNA-binding domain. This domain is characterized by the structure of a Cys2/Cys2(C2/C2) zinc finger binding specifically to the cis-regulatory element comprising the common core sequence (AT)/AAAG [1–2]. The DNA-binding domain, which binds DNA and interacts with other proteins, is a vital functional domain [3]. The C-terminus of the Dof proteins contains a transcriptional regulation domain with various functions, including interaction with diverse regulatory proteins and the activation of gene expression [4]. The N- and C-terminal regions of the Dof protein may interact with diverse regulatory proteins or intercept signals to mediate the activation or repression of the target genes [5].

The *Dof* genes are ubiquitous in many plant species and are plant-specific transcription regulators that are involved in various abiotic processes. The DOF protein (*ZmDOF1*) first identified in maize plays a role in light-regulated gene expression, but no *Dof* genes have been isolated from other eukaryotes, such as yeast or humans [5]. In *Arabidopsis*, some of the well characterized *Dof* genes were shown to be involved in many plant biological processes. For example the *DAG1* genes had the active involvement in seed germination [6]; the *CDF1*, *CDF2*, *CDF3* and *CDF5* genes are associated with photoperiodic regulation of flowering [7–8]; and *HPPBF3*, *COG1* and *OBP3*, participate in the regulation of phytochrome signaling [9–11]. In rice, *OsDof3* regulates the expression of gibberellins, *OsDof12* and *OsDof23*, regulate flowing time and seed expression, respectively, and *OsDof24* and *OsDof25* are involved in carbon and nitrogen metabolism [12–13]. In tobacco, the *Sar8*.*2b* gene can be activated by the Dof transcription factor, which is related in systemic acquired resistance [14]. In sorghum, *SbDof* genes are associated with the responsiveness to light, hormones and endosperm-specific genes [15]. In maize, *Dof1* plays a regulatory role in controlling gene related to carbohydrate metabolism [2]. In wheat, *TaDof1* is related to carbon metabolism by increasing the regulation of the C4 pathway [16]. *WPBF* of wheat is involved in growth and development processes [17]. In addition, *Dof* genes are involved in physical interactions with other TFs such as bZIP, MYB, and WKRY, implying they have been implicated in the regulation of plant physiological processes.

To date, few studies are concerned with *Dof* gene family in *P*. *trichocarpa*, compared with the comprehensive researches of these genes in other plant species. Moreover, previous research rarely mentioned the responses of *Populus Dof* genes to drought stress. Therefore, there dose require a comprehensive analysis of the *Dof* gene family in *P*. *trichocarpa*. In this study, 41 *Dof* genes were systematically studied, along with their gene structures and promoter *cis*-elements. Their expression profiles in the leaves and roots of *Populus* under drought stress were examined using heatmap data and gene expression analysis with quantitative real-time reverse transcription polymerase chain reaction (qRT-PCR) analyses under osmotic and abscisic acid (ABA) treatments. Our study is the first time to provide insight about the role of *PtrDof* genes in stress response in *Populus*.

*Populus trichocarpa* is a valuable forest resource used widely to produce various paper-based and timber products, and has genuine commercial and ecological value. However, recent drought induced forest mortality became more and more serious, especially for Poplar. Thus, identifying *PtrDof* genes will provide a novel insight for drought stress resistance in *Populus*.

## Materials and Methods

### Identification and characteristics of the *Dof* gene family

Data for the *P*. *trichocarpa* genome, including the protein database and the genomic and cDNA libraries, were obtained from the Phytozome v9.1 (http://www.phytozome.net/search.php) and NCBI (http://www.ncbi.nlm.nih.gov/) databases. We searched for the *Dof* genes of *P*. *trichocarpa* using two verified methods. The first method was using the Protein family (Pfam 27.0) database (http://pfam.sanger.ac.uk/), the second method was using the Hidden Markov Model (HMM) profile of the *Dof* gene family (protein family ID: PF02701). We verified all of the located sequences by additional manual analysis to ensure a zf-Dof domain does exist with the SMART (http://smart.emblheidelberg.de/) database [18]. All available genes were contrasted with the *Dof* gene family in PlnTFDB v3.0 (http://plntfdb.bio.uni-potsdam.de/v3.0/) to avoid missing genes [19]. Any additional genes were analyzed further. The ExPasy (http://web.expasy.org/protparam/), an online program was used in calculating the molecular weight and isoelectric point (pI) [20]. The subcellular localization of the *Dof* genes was predicted with WoLF PSORT (http://wolfpsort.org/) [21].

### Phylogenetic analysis, exon/intron structure analysis and identification of conserved motifs

Multiple alignments were made using the Clustal X program (version 1.83), on the basis of the protein sequences [22]. The neighbor-joining approach was used to construct unrooted phylogenetic trees in MEGA 5.0 [23]. The exon/intron organizational analyses were revealed with the Gene structure display server (GSDS 2.0, http://gsds.cbi.pku.edu.cn/index.php) [24]. Conserved motifs were analyzed with the Multiple Expectation Maximization for Motif Elucidation (MEME) (Version 4.9.1, http://meme.nbcr.net/meme/) [25].

### Chromosomal location

The chromosomal locations of genes were mapped by the PopGenIE v3 database (http://www.popgenie.org/) [26]. A physical map was constructed with Adobe Illustrator CS5 (Adobe Systems Incorporated). Genes are separated by five or fewer gene loci with a 100 kb distance were defined as tandem duplicates [27].

### Promoter cis-element analysis

We obtained the promoter sequences of all *Dof* genes from the Phytozome v10.0 database and analyzed them using the cis-element database PlantCARE (http://bioinformatics.psb.ugent.be/webtools/plantcare/html/) [28], thus predicting and presenting cis-acting regulatory elements.

### Gene Ontology (GO) annotation

Blast2GO v3.0 was used to analyze the functional classfication of *Dof* sequences and obtain the details of the annotation results [29]. Genes are described according to three classifications of GO categories: biological processes, molecular functions and cellular components.

### ExHeatMap analysis

The PopGenIE v3 database (http://www.popgenie.org/) was used to obtain the expression profiles of *Populus Dof* genes in leaves and roots under drought stress. Detailed descriptions of *Dof* gene expression in response to drought stress were downloaded from the exPlot tool at the PopGenIE v3 database. Cluster and java were used to analyze these data.

### Plant material and treatment

*P*. *trichocarpa* (genotype Nisqually-1) was donated by professor Liquan Jiang of North Carolina State University. *P*. *trichocarpa* was clonally propagated by culture in half-strength Murashige and Skoog medium (1/2 MS, pH 5.8) under long-day conditions (16 h light / 8 h dark) at 25°C. Before determining the final concentration of mannitol, we performed the preliminary experiment to evaluate the effect of different concentrations of mannitol on plants growth *in vitro*. The results showed that 150 mM mannitol could reduce but not abolish growth of plants *in vitro*. For osmotic stress, plantlets were exposed to 150 mM mannitol. Plants cannot take in water normally due to the high osmotic pressure of mannitol. Thus, the mannitol simulated osmotic stress by increasing osmotic shock. For ABA stress conditions, plantlets were treated with 200 μM ABA. Mannitol was added to 1/2 MS medium at the stated concentrations and then autoclaved (115°C, 15 min). ABA was filter-sterilized and then mixed into 1/2 MS medium at the stated concentrations. The 150 mM mannitol and 200 μM ABA treatments lasted for 0, 3, 6, 12, or 24 h or 7 d. Young leaves and roots were collected as samples at all time points. Each stress treatment performed with three biological replicates. All samples were quickly frozen in liquid nitrogen and stored at −80°C until using. Untreated plants were used as controls.

### RNA isolation and qRT-PCR verification

Total RNA was extracted using the cetyltrime thylammonium bromide method from roots and leaves [30]. The cDNA synthesized using the TransScript^®^ One-Step gDNA Removal and cDNA Synthesis SuperMix. The specific sequences of each gene were based on results from multiple sequence alignment (http://multalin.toulouse.inra.fr/multalin/multalin.html). The specific primers for *Dof* genes were designed by Primer Premier 5 according to its CDS with following parameters, melting temperatures of 58–62°C, primer lengths of 18–22 bp and product lengths of 190–210 bp. The primer details are listed in S1 Table.

QRT-PCR was carried out using a TransStart^®^ Top Green qPCR SuperMix (TransGen Biotech, Beijing, China) to determine the transcript levels under stress. Reactions were performed in 20 μL volume containing 10 μL 2×TransStart^®^ Top Green qPCR SuperMix, l μL cDNA template, 7 μL ddH_2_O, and 2 μL each primer-specific. Three experimental replicates were used for each sample to maintain accuracy. The *P*. *trichocarpa Actin1* gene (GenBank ID: XM_002298674) was used as the reference gene [31]. Other specifications, including PCR conditions and relative gene expression calculations we based on previous study [32]. All reactions were carried out under the following PCR conditions: an initial denaturation step of 95°C for 1 min, a three-step thermal cycling profile of denaturation at 95°C for 5s, primer annealing at 55°C for 30 s, and extension at 72°C for 30 s. Then, an additional step of 80°C for 1 s was performed to remove primer dimers, followed by plate reading. We used the relative quantification method (2^−ΔΔCT^) to evaluate relative gene expression between replicates.

### Statistical analysis

All the statistical analysis used one-way ANOVA to determine significance. Tukey’s test was analyzed to compare the difference. Significance was defined as * *P* < 0.05, ** *P* < 0.01.

### miRNA target analysis and target validation by RLM-RACE

PMRD (http://bioinformatics.cau.edu.cn/PMRD) was used to download the mature *P*. *trichocarpa* miRNA sequences. The Plant Small RNA Target Analysis Server (psRNA Target: http://plantgrn.noble.org/psRNATarget) was used to identify the miRNA target genes in the *PtrDof* families with default parameters. FirstChoice RLM-RACE Kit (Invitrogen, Thermo Fisher Scientific) was used to perform RLM-RACE and illustrate the predicted targets following the methods researched by Song et al [33]. The gene-specific primers for RLM- RACE are presented in S2 Table. The RLM-RACE products were ligated into the pMD18-T vector (TaKaRa), and sequenced.

## Results

### Identification of *Dof* genes in *Populus*

We used the HMM profile of the Pfam Dof domain (protein family ID: PF02701) to query the *Dof* genes in the *P*. *trichocarpa* genome. All the obtained *Dof* genes were checked using the SMART database to ensure the presence of the Dof domain. Then, 41 *Dof* genes were identified and was close to results from various biology analyses which estimated 30, 36, 24, 31 and 54 *Dof* genes in rice, *Arabidopsis*, barley, wheat and maize, respectively [34–36].

Molecular masses of the *Dofs* in *P*. *trichocarpa* varied from 17735.9 to 55263.5 Da. The encoded proteins ranged from 159 to 1485 amino acids (aa), with an average of 684 aa. The location of the protein expression levels in the plant cell were predicted with WoLF PSORT. The majority *Dof* genes were predicated as nuclear proteins, except *PtrDof23*, which was located in the mitochondrial matrix (Table 1).

**Table 1:** The Dof gene family in *Populus trichocarpa*.

Gene name	Accession number	NCBI locus ID	Length (aa)	MW (Da)	pI	Localization^a^
***PtrDof1***	POPTR_0004s05580	XM_002305034.2	159	17735.9	9.23	nucl
***PtrDof2***	POPTR_0004s04590	XM_002305748.1	325	35553.0	9.15	nucl
***PtrDof3***	POPTR_0005s21130	XM_002307448.2	274	30875.5	5.07	nucl
***PtrDof4***	POPTR_0005s14080	XM_002306417.1	331	35465.6	9.51	nucl
***PtrDof5***	POPTR_0005s19310	XM_002307281.1	342	37004.9	8.81	nucl
***PtrDof6***	POPTR_0007s11620	XM_002310717.2	323	34168.9	8.96	nucl
***PtrDof7***	POPTR_0011s07400	XM_002316790.2	165	18292.5	8.94	nucl
***PtrDof8***	POPTR_0019s05720	XM_002325405.2	493	53501.1	5.50	nucl
***PtrDof9***	POPTR_0015s03520	XM_002322008.2	321	35235.4	7.21	nucl
***PtrDof10***	POPTR_0015s08810	XM_002321564.1	314	34797.7	6.19	nucl
***PtrDof11***	POPTR_0016s07000	XM_002322747.1	225	25219.8	6.23	nucl
***PtrDof12***	POPTR_0011s05410	XM_006377318.1	325	35711.2	9.26	nucl
***PtrDof13***	POPTR_0011s05450	XM_006377322.1	357	39200.7	8.97	nucl
***PtrDof14***	POPTR_0010s21240	XM_006378649.1	356	37530.7	9.33	nucl
***PtrDof15***	POPTR_0014s03590	XM_006374952.1	261	27530.1	5.95	nucl
***PtrDof16***	POPTR_0002s07150	XM_002300897.2	301	34180.8	4.71	nucl
***PtrDof17***	POPTR_0002s13100	XM_006386451.1	306	32229.4	5.57	nucl
***PtrDof18***	POPTR_0006s21700	XM_002309218.1	288	31912.0	6.29	nucl
***PtrDof19***	POPTR_0012s12670	XM_002317912.2	329	36234.4	6.36	nucl
***PtrDof20***	POPTR_0012s02570	XM_002317757.2	297	32791.0	7.69	nucl
***PtrDof21***	POPTR_0012s08280	XM_002318016.1	312	34205.1	6.43	nucl
***PtrDof22***	POPTR_0004s12120	XM_002305996.2	503	55067.6	5.38	nucl
***PtrDof23***	POPTR_0007s11790	XM_002310726.2	248	25507.4	8.57	mito
***PtrDof24***	POPTR_0013s06290	XM_002319159.2	1485	53797.4	6.57	nucl
***PtrDof25***	POPTR_0017s12080	XM_002323827.2	506	55263.5	5.42	nucl
***PtrDof26***	POPTR_0007s09520	XM_006380521.1	494	37080.9	8.28	nucl
***PtrDof27***	POPTR_0004s03900	XM_002305645.2	304	33910.7	8.73	nucl
***PtrDof28***	POPTR_0011s04730	XM_002316595.2	305	33893.5	8.41	nucl
***PtrDof29***	POPTR_0014s09640	XM_002320172.2	229	25083.8	9.20	nucl
***PtrDof30***	POPTR_0003s02890	XM_006385272.1	235	25146.4	8.96	nucl
***PtrDof31***	POPTR_0003s14450	XM_002303642.2	279	30688.2	8.63	nucl
***PtrDof32***	POPTR_0001s24540	XM_002299843.1	332	35591.7	9.58	nucl
***PtrDof33***	POPTR_0002s17490	XM_002301384.2	263	28908.9	8.99	nucl
***PtrDof34***	POPTR_0005s13990	XM_002306412.2	253	25995.8	8.83	nucl
***PtrDof35***	POPTR_0006s08440	XM_002308117.2	326	34632.6	9.10	nucl
***PtrDof36***	POPTR_0001s11130	XM_002299405.1	285	31392.1	8.42	nucl
***PtrDof37***	POPTR_0008s08740	XM_006379606.1	500	54067.2	6.51	nucl
***PtrDof38***	POPTR_0010s17480	XM_002316129.2	496	54199.0	6.91	nucl
***PtrDof39***	POPTR_0008s05520	XM_002311128.2	345	36903.3	9.13	nucl
***PtrDof40***	POPTR_0009s03490	XM_002314153.1	326	34651.5	9.33	nucl
***PtrDof41***	POPTR_0015s01160	XM_002321946.2	255	28121.2	8.78	nucl

^a^ *nucl* nuclear; *mito* mitochondrial matrix

### Phylogenetic analysis, exon/intron structure analysis and identification of conserved motifs

**Fig 1:**
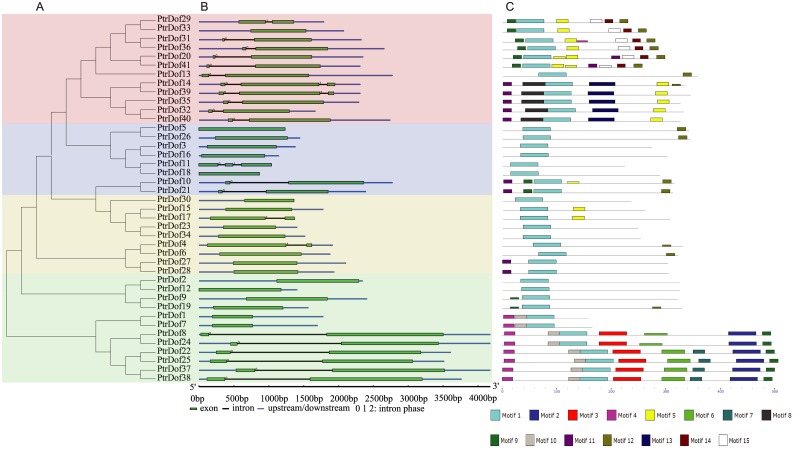
Phylogenetic relationships, gene structure and motif compositions of *Populus Dof* genes. A. Multiple alignment of 41 full-length amino acid sequences of *Populus Dof* genes, executed by ClustalX 1.83. The phylogenetic tree was constructed using MEGA 5.0 and the neighbor-joining method. Support values from a bootstrap analysis with 1,000 replicates are specified at each node. The four major phylogenetic subgroups are marked with different colored backgrounds. B. Exon/intron structures. Exons and introns are represented by particular colored boxes and black lines, respectively. C. Schematic representation of the conserved motifs identified by MEME. Each colored box represents a motif and black lines represent non-conserved sequences.

To examine the phylogenetic relationships, an unrooted phylogenetic tree was drawn (Fig 1A). The 41 *PtrDof* homologs were separated into four groups (A to D). Subgroup A and D constituted the largest clade with 12 members each, subgroup B contained the fewest *PtrDof* family members (8 genes), and group C contained 9 members.

To check the structural diversity, we investigated the characterization of exon-intron structure in the genomic DNA sequences of individual *PtrDof* genes (Fig 1B). The predicted numbers of exons among the *PtrDof* genes were relatively fewer, varying from one to three, with 19 members having one and 19 with two. Three (*PtrDof14*, *PtrDof39* and *PtrDof11*) genes had three exons. Furthermore, some *PtrDof* genes belonging to the same subgroup had similar gene structures, such as introns numbers and extons lengths. For instance, subgroup A genes had one or two introns expect *PtrDof33*, and subgroup C genes had zero introns with exception of *PtrDof17* and *PtrDof 4*. The majority of three-exon-genes belonged in subgroup A. These similar structural features may be related to their functions in the *Populus* genome (Fig 1B).

MEME programmer was used to assess PtrDof proteins to characterize motif compositions (Fig 1C). A total of 15 conserved motifs were identified (S3 Table). These motifs are shown in the location of corresponding protein. The majority of related members in the phylogenetic tree had motifs. All genes uniformly contained common motif 1 at the C-terminal region, which was confirmed to be a conserved Dof domain. Moreover, differences in gene composition and motif organization among related *PtrDofs* members within the same subgroups indicated that these genes may have a divergent function.

### Chromosomal location and gene duplication

**Fig 2:**
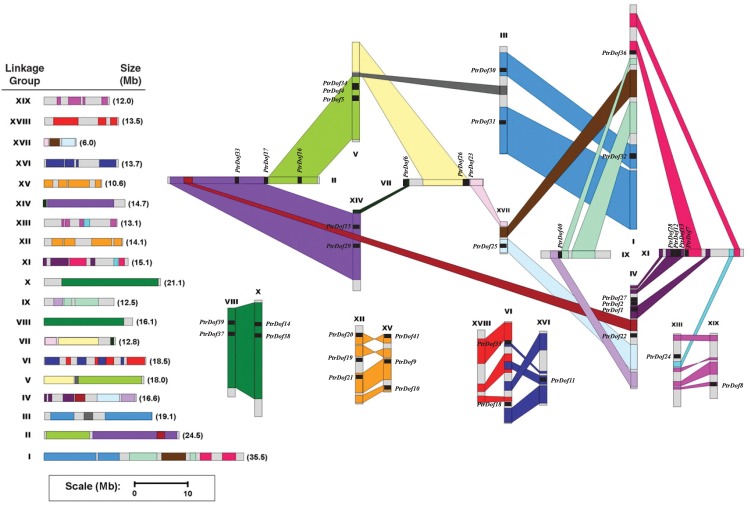
Chromosomal locations of *Populus Dof* genes. Forty-one *Dof* genes were mapped to 19 linkage groups (LG). A schematic view of chromosome reorganization caused by recent whole-genome duplication in *Populus* is shown. Segmental duplicated homologous blocks are indicated by the same color. The scale represents mega bases (Mb). The LG numbers are indicated above each bar.

In silico mapping of the gene loci indicated that the distribution of *PtrDof* genes span all 19 linkage groups (LGs) in an uneven manner. For example, *PtrDof3* was located in the chromosomal scaffold that was not marked in the figure. As shown in Fig 2, the 41 *PtrDof* genes were dispersed on 18 of the 19 *Populus* chromosomes (none in chromosome XVIII). Chromosome IV and XI harbored the most (4 of 41) genes. On contrast, only one gene was found on each of chromosome IX, XIII, XVI, XVI I, and XIX. The rest of the chromosomes harbored two or three genes.

Researches previously showed that the *Populus* genome has gone through three circles of genome-wide duplication at any rate including multiple segmental duplications, tandem duplications and transposition events in that order [37]. The segmental duplication associated with the salicoid duplication event that happened 65 million years ago promoted the expansion of numerous multigene families [27, 38–40]. We mapped the *PtrDof* genes to the duplicated blocks based on the previous research to check sure the possible relationship between the *Dof* genes and segmental duplications. The distribution of genes associated with the corresponding duplicate blocks is demonstrated in Fig 2. Approximately 49% (20 of 41) genes were firstly located in duplicated regions. Twelve duplicated genes (*PtrDof1*, *6*, *8*, *10*, *11*, *12*, *13*, *23*, *25*, *26*, *28* and *31*) were only contained in one of the blocks and lacked duplicates in the corresponding block. While, eight genes were located outside any duplicated blocks. The segmental duplication also occurred in subgroup C. These results suggest that the *PtrDof* genes likely originated from both segmental and tandem duplications.

### Promoter *cis*-element analysis

Phytohormones such as salicylic acid (SA), jasmonic acid (JA), ethylene (ET), and ABA are involved in various processes throughout plants to accommodate abiotic stresses. We identified the putative *cis*-acting regulatory DNA elements by analyzing the promoter sequences of all *PtrDof* genes. In the *PtrDof* gene promoter region, many key *cis*-elements that were related to environmental stress signal responsiveness were identified, such as MBS (MYB binding site, involved in drought-inducibility), HSE (heat stress-responsive element), C-repeat/DRE (cold and dehydration-responsive element), TC-rich repeats (defense and stress-responsive element), LTR (low temperature-responsive element), and W-Box (WRKY binding site, involved in abiotic stress responsiveness). Other key elements included those in phytohormone signaling, such as ABRE (abscisic acid-responsive element), ERE (ethylene-responsive element), TCA-element (salicylic acid-responsive element), CGTCA-motif (MeJA-responsive element), TGACG-motif (MeJA-responsive element), and P-box (gibberellin-responsive element) (S4 Table). Most of the *PtrDof* genes containing *cis*-elements contained responsiveness to phytohormone signaling and environmental stress signal, whereas *PtrDof18* and *41* contained only three cis-elements (S5 Table).

### Gene Ontology (GO) annotation

**Fig 3:**
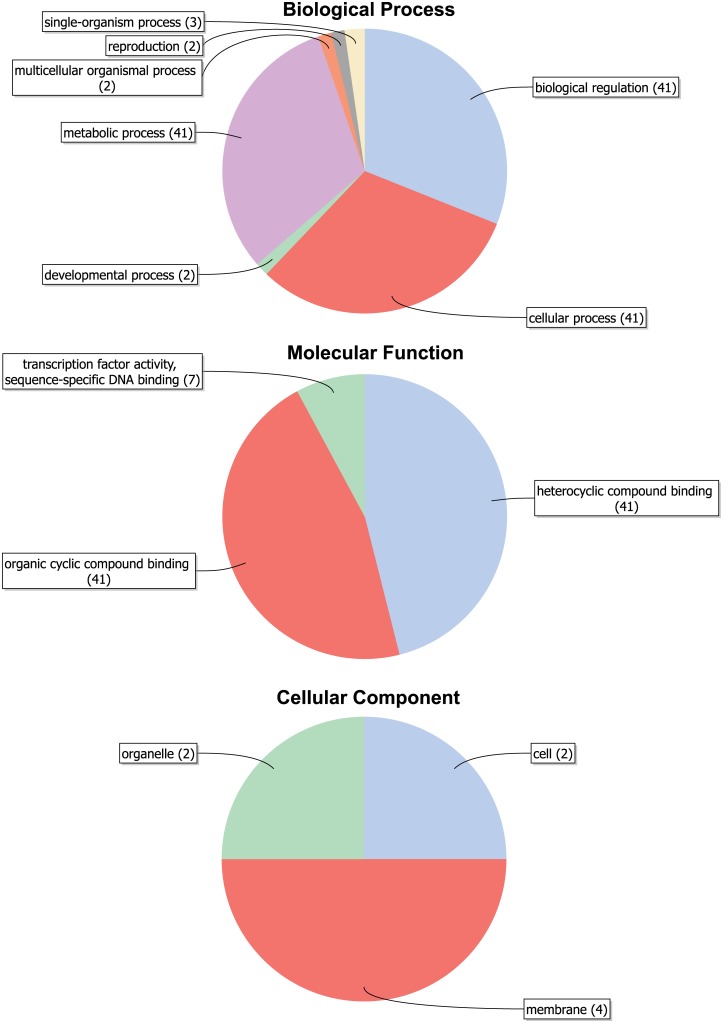
Gene Ontology (GO) results for *Populus* Dof proteins. GO analysis of 41 *Dofs* sequences predicted for their involvement in biological processes, molecular functions and cellular components. The results are presented as detailed bar diagrams in S6 Table.

The 41 *PtrDof* genes were classified into biological processes, molecular functions and cellular components by Gene Ontology (GO) using Blast2GO v3.0 (Fig 3, S6 Table). The seven terms of biological processes were defined. The function of all *PtrDofs* were predicated in the metabolic process, the cellular process and the biological regulation process, followed by the single-organism (~7%) and the multicellular organismal processes and reproduction or developmental process (~5%). Molecular function predictions showed that all *PtrDofs* were in accordance with organic cyclic compound binding and heterocyclic compound binding. In addition, some *PtrDofs* were annotated as sequence-specific DNA binding involved in transcription factor activity (~17%). Furthermore, cellular component prediction indicated that four *PtrDofs* were localized in the membranes (~10%). Only two *PtrDofs* were organelle-localized and cell-localized (~5%).

### Expression profiles under drought stress

**Fig 4:**
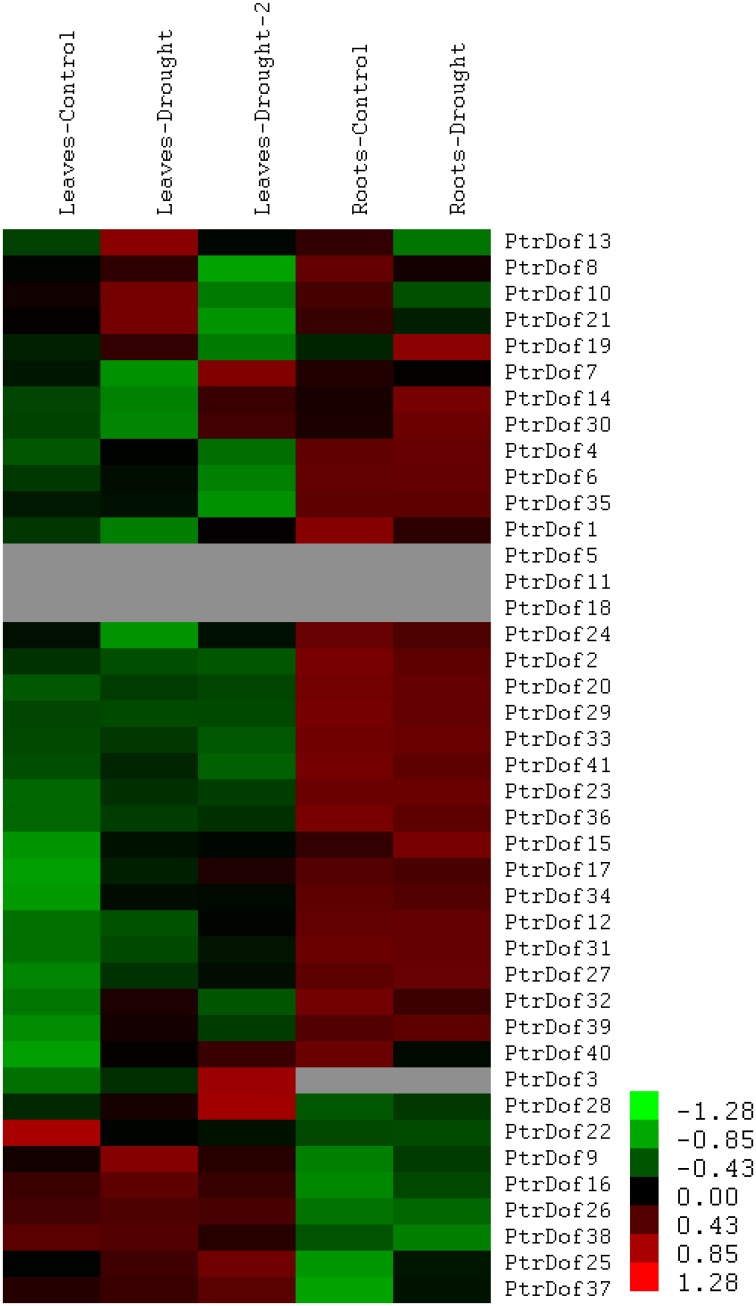
Expression profile of *Populus Dof* genes under drought stress. The heatmap was visualized using the exPlot tool in the PopGenIE v2 database. Heatmap showing 41 *Populus Dof* genes in leaves and roots under drought stress.

A majority of land plants encounter environmental stress during their life span and drought is the major environmental stress. In order to characterize the possible roles of *PtrDof* genes in drought stress, we used the publicly available data to investigate the expression profiles of *PtrDof* genes responded to drought stress. A detailed description of gene expression was downloaded from the exPlot tool at the PopGenIE v3 database. The data for all the *PtrDof* genes are shown in S7 Table, except for *PtrDof5*, *11*, *18*, and *PtrDof3*, for which the root has no data. 31 genes were upregulated in leaves and 14 genes were upregulated in roots under drought stress (Fig 4). Ten genes were all upregulated in leaves and roots.

### Expression pattern of *PtrDof* genes under osmotic and ABA stress

**Fig 5:**
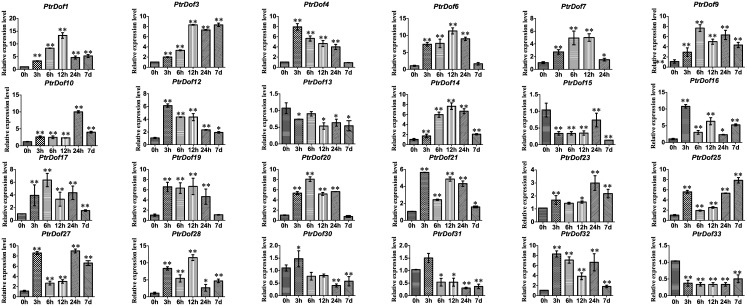
Expression analysis of 31 selected *PtrDof* genes in leaves under osmotic stress using qRT-PCR. The relative mRNA abundance of 31 selected *PtrDo****f*** genes was normalized with respect to the reference gene (*Actin1*). Error bars represent the standard deviations of three biological replicates. Asterisks indicate stress treatment groups that showed a significant difference in transcript abundance compared with the control group (* P < 0.05, ** P < 0.01).

**Fig 6:**
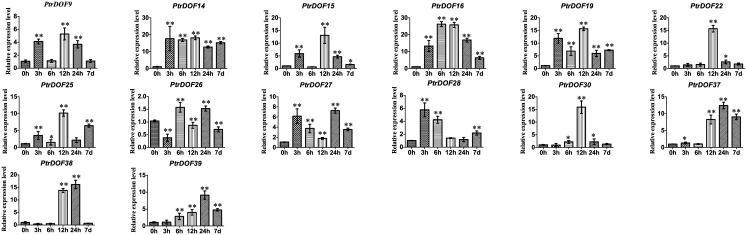
Expression analysis of 14 selected *PtrDof* genes in roots under osmotic stress using qRT-PCR. The relative mRNA abundance of 33 selected *PtrDo****f*** genes was normalized with respect to the reference gene (*Actin1*). These genes were upregulated in roots under drought stress according to the HeatMap. Error bars represent the standard deviations of three biological replicates. Asterisks indicate stress treatment groups that showed a significant difference in transcript abundance compared with the control group (* P < 0.05, ** P < 0.01).

**Fig 7:**
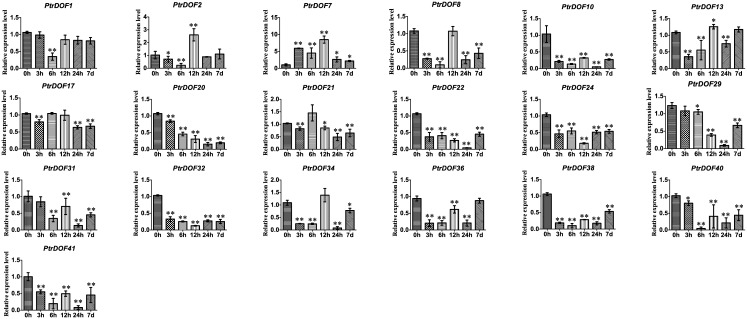
Expression analysis of 19 selected *PtrDof* genes in roots under osmotic stress using qRT-PCR. The relative mRNA abundance of 19 selected *PtrDo****f*** genes was normalized with respect to the reference gene (*Actin1*). These genes were downregulated in roots under drought stress according to the HeatMap. Error bars represent the standard deviations of three biological replicates. Asterisks indicate stress treatment groups that showed a significant difference in transcript abundance compared with the control group (* P < 0.05, ** P < 0.01).

Numerous *PtrDof* genes were obtained by heatmap analysis with differences in the expression profiles under drought stress. To verify these results, qRT-PCR was used to study the differential expression of selected genes under osmotic stress. For the leaves, 24 genes were induced, 4 genes were suppressed, and 3 genes were irregular (Fig 5). The results were broadly consistent with the heatmap data, which means the drought-induced genes were all upregulated and the drought-suppressed were all downregulated in leaves. For the roots, 15 genes were induced and 18 genes were suppressed (Figs 6 and 7). A total of 15 genes (*PtrDof1*, *8*, *10*, *17*, *20*, *21*, *22*, *24*, *29*, *32*, *34*, *36*, *38*, *40* and *41*) were significantly downregulated at all time points, while only 8 genes (*PtrDof14*, *16*, *19*, *25*, *27*, *28*, *37* and *39*) were upregulated at all time points. The expression tendencies were roughly consistent with the exPlot analysis. In addition, *PtrDof14*, *16*, *25*, *27*, 28, *37* and *39* were all upregulated in the leaves and roots under osmotic stress (Figs 5, 6 and 7).

**Fig 8:**
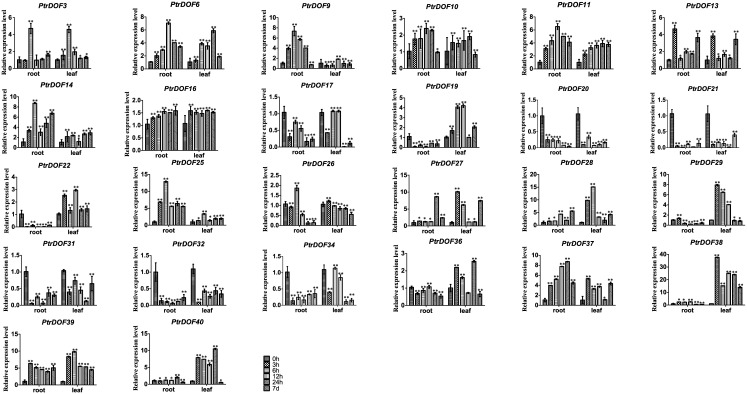
Expression analysis of 26 selected *PtrDof* genes in leaves and roots under ABA stress qRT-PCR. The relative mRNA abundance of 26 selected *PtrDo****f*** genes was normalized with respect to the reference gene (*Actin1*). Error bars represent the standard deviations of three biological replicates. Asterisks indicate stress treatment groups that showed a significant difference in transcript abundance compared with the control group (* P < 0.05, ** P < 0.01).

We got putative cis-acting elements by analyzing the promoter sequences of all *PtrDof* genes. Twenty-six *PtrDof* genes harbored ABRE in their promoter region. QRT-PCR was used to analyze the expression profiles under ABA stress (Fig 8). In leaves, 16 genes were upregulated, 8 genes were broadly downregulated and two genes were not influenced by ABA. In roots, 15 genes were upregulated and 11 genes were downregulated. In leaves, 13 genes (*PtrDof3*, *10*, *11*, *13*, *14*, *16*, *22*, *25*, *27*, *28*, *37*, *38*, *39* and *40*) were upregulated significantly within a short time (24 h), whereas 12 genes (*PtrDof1*, *6*, *10*, *11*, *13*, *14*, *16*, *25*, *27*, *28*, *37*, *39* and *40*) were upregulated significantly within a short time (24 h) in roots. However, among these genes, most were also upregulated in the long term (7 d), except *PtrDof10* and *40*. *PtrDof11*, *13*, *14*, *16*, *25*, *27*, *28*, *37* and *39* were all upregulated at all points in leaves and roots under ABA. A total of 7 genes (*PtrDof14*, *16*, *25*, *27*, *28*, *37* and *39*) in the *Populus Dof* gene families were upregulated at every time points in the leaves and roots under osmotic and ABA stress (Figs 5, 6, 7 and 8).

### MiRNA target site prediction and validation

**Fig 9:**
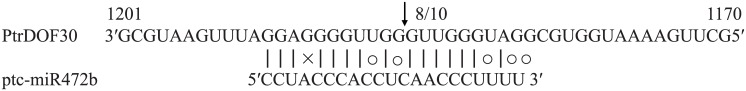
Mapping of mRNA cleavage sites confirmed by 5′ RLM-RACE. Arrows indicate the 5′ ends of the mRNA fragments, as identified by cloned 5’ RLM-RACE products, with the frequency of clones shown.

Among the *PtrDof* genes, 12 were targeted by 15 miRNAs. *PtrDof12*, *PtrDof13* and *PtrDof40* were targeted by two miRNAs (S8 Table). *PtrDof12* was targeted by ptc-miRf11023-akr and ptc-miRf12020-akr. *PtrDof13* was targeted by ptc-miRf10053-akr and ptc-miRf10957-akr. *PtrDof40* was targeted by ptc-miRf10540-akr and ptc-miRf11148-akr. The other *PtrDofs* were only targeted by one miRNA. The sequence analysis of *PtrDof30* implied that the 1170–1190 bp region may be the target site of ptc-miR472b. Ptc-miR472b miRNA was previously implicated with stress response [41]. *PtrDof 30* was confirmed as a real target of miRNA, as all of the 5′ ends of the mRNA fragments mapped to the nucleotide that paired to the tenth nucleotide of each miRNA with higher frequencies than depicted for each pairing oligomer (Fig 9).

## Discussion

The *Dof* genes are ubiquitous in many plant species and are specific plant transcription regulators that are involved in various abiotic stress responses. The functional and evolutionary analysis of *Dof* genes have been preliminary performed in *Arabidopsis*, rice, soybean, Chinese cabbage, potato, pigeonpea, tomato and cucumber. In this report, we conducted a comprehensive analysis of the *PtrDof* family in *P*. *trichocarpa* to confirm their potential functions in response to osmotic stress.

We identified 41 putative full-length *PtrDof* genes in the *P*. *trichocarpa* genome. The number of *PtrDof* homologues was similar to that in *Arabidopsis* and rice [42]. The lengths of these sequences varied, which implied a highly complicacy among the *PtrDof* genes. Approximately 98% (40 of 41) of the *PtrDof* genes were identified to be localized in the nuclear, however only one gene was identified as the mitochondrial matrix (Fig 1A). The results were same as a previous analysis of the *Dof* gene family in cucumber where CsDof proteins were predicted as the nucleus except for *CsDof30*, which was extracellular [43].

Similar to previous discoveries in rice and *Arabidopsis* [42], the *PtrDof* genes had few introns (0–2) in each gene (Fig 1B). The motif analysis showed that motif 1 was uniformly observed in all Dof proteins (Fig 1C), similar to *Arabidopsis*, rice and tomato. This result indicated that the evolution of PtrDof transcription factors was conserved in plant development.

With high contribution to genetic novelty, the whole-genome, tandem and segmental duplications are important for genomic expansion, as the fundamental sources of genetic novelty [44–45]. We observed that 78% of *PtrDof* genes appeared to contain duplicated regions; however, eight genes were located away the duplicated blocks (Fig 2). The results suggested that partial genomic duplication included rearrangements, which resulted in the loss of a number of genes. The present study found only one pair (*PtrDof4/34*) with tandem duplication and ten pairs of segmental duplication events in *PtrDof* genes. This indicated that *PtrDof* gene segmental duplication and not tandem duplication is predominantly involved in the evolution of *P*. *trichocarpa*. The result was similar to that observed for *CsDof* duplications in cucumber [43].

The *cis*-elements play vital parts in the transcriptional regulation of gene expression, controlling phytohormone responses and complicated abiotic stress, to increase the resistance of plants under fluctuating environments. In our report, many *cis*-elements related to abiotic stress and phytohormone have been identified, including ABRE, MBS, HSE, ERE and TCA-elements (S4 Table). Every gene in the family included three *cis*-elements mentioned at least (S5 Table). *PtrDof18* and *41* have only three *cis*-elements, which indicated that these genes might not be related to abiotic stress. By comparison, *PtrDof11*, *16*, *19* and *36* have nine *cis*-elements, which indicated that these genes might be strongly associated with functions under different abiotic stresses. According to the expression profiles of the *Populus Dof* genes under various stresses, *PtrDof16*, *PtrDof19* and *PtrDof36* were upregulated in leaves at some time points after osmotic and ABA treatment. These results were roughly consistent with the promoter analysis.

The Dof family of transcription factors is a major large class of plant-specific factors, which have been identified to be involved in the regulatory networks of plants as very critical roles in response to abiotic stress. The exPlot data indicated that *PtrDofs* have important functions in drought stress. Under osmotic conditions, a total of 7 genes (*PtrDof14*, *16*, *25*, *27*, *28*, *37* and *39*) in the *Populus Dof* gene family were all upregulated at all time points, including short and long term, in the leaves and roots (Figs 5, 6, 7 and 8). These results further suggest the involvement of these genes in osmotic stress as well as long-term response genes to osmotic stress. An *Arabidopsis* homolog (*AT5G66940*) of two of the upregulated genes (*PtrDof27* and *28*) was similarly upregulated under drought conditions [46]. Under ABA conditions, *PtrDof27* and *28* and their homolog gene (*AT5G66940*) also have similar responsive expression in the leaves and roots, which were both induced [46]. Moreover, in response to osmotic treatment, osmotic upregulated *PtrDof* members grouped into four subgroups in the leaves. In Chinese cabbage, the 9 *BraDof* genes from different classes were all induced by drought stress in the leaves [4]. However, in Triticum, only two *TaDof* genes from two different clades were significantly upregulated by drought [36]. Probably, the mechanisms of *Dof* genes in response to abiotic stress may be various in different plant species [4]. We found that eight genes were upregulated under osmotic and ABA treatments in roots. The results indicated that *PtrDofs* may be involved in the development of plant roots. A previous study found that *Populus Dofs* were associated with the formation of adventitious roots [47]. Different *PtrDof* genes had different expression pattern under the same conditions. Our research illustrates that PtrDof transcription factors act as a vital role resistance to osmotic stress in plants.

The miRNAs act as a vital role during plant development and abiotic stresses-response. We found that 12 *PtrDof* genes were targeted by 15 miRNAs. Each miRNA acts on different genes in most cases. It was indicated that 16 miRNA families could target 13 *CmDOF*s previously and there was no repeat among these miRNAs [43]. The results indicated that each miRNA has its specific regulatory genes and that one miRNA may not simultaneously act on different *Dofs*. Four genes, including *PtrDof12*, *PtrDof13* and *PtrDof40*, were targeted by two miRNAs, whereas the other nine genes only were targeted by one miRNA. Among the *Dof* gene family in the cucumber, there were three target sites for *CmDOF1* and two target sites for *CmDOF21*,whereas the remaining 11 *CmDOFs* have only one target site [43]. In plants, single or multiple miRNAs are induced to regulate the expression of target genes under various stresses to improve the adaptability of plants [48]. The 12 *PtrDof* target genes belong to four groups of *PtrDofs*. Subgroup D contains the most target genes (four), whereas subgroup B only contains one target gene. Subgroup A and C, respectively, contain four and two target genes. In the cucumber, each subgroup also has a different number of target genes [43]. MiR472 is a miRNA induced by different stresses. The recent study showed that miR472 acts as a negative regulator, preventing an autoimmune response that would have detrimental consequences on plant fitness in *Arabidopsis* [49], and the targets of miR472 were identified as disease resistance genes in *Arabidopsis lyrata* [50]. The expression of miR472 plays a role in influencing the expression of their target genes, which are involved in disease resistance in *Citrus sinensis* [51]. The ptc-miR472b miRNA is regulated by cold stress in *Populus* [41], but no definite results are available. The expression of miR472 has been shown in the PMRD and the expression in rice was decreased under oxidase stress. The gene expression omnibus of NCBI has reported that miR472 had different expression in an experiment designed by Zhongs et al (http://www.ncbi.nlm.nih.gov/geo/query/acc.cgi?acc=GSE11535). However, to date, the interactions between miR472 and osmotic stress-related targets have not been reported. Our study provides a new target gene, *PtrDof30*, which is a osmotic-related gene in *P*. *trichocarpa*. We also confirmed that *PtrDof 30* was a real target of miRNA in plants (Fig 9).

## Conclusions

Dof-family transcription factors were comprehensively analyzed in the genome of *Populus trichocarpa*. We performed 41 *PtrDof* genes that were classified into four groups. Most genes within the same group had similar gene structures and conserved motifs. *PtrDofs* were dispersed on 18 of the 19 *Populus* chromosomes and likely originated from both tandem and segmental duplications. Promoter *cis*-element analysis indicated that most *PtrDof* genes contain *cis*-elements in response to stress and phytohormones. The HeatMap data for the *PtrDof* genes suggest that they are primarily expressed in leaves and roots. The expression of selected *PtrDof* genes was characterized in response to osmotic and ABA stresses and indicated that *PtrDofs* may be involved in resistance to abiotic stress in *Populus trichocarpa*. In addition, 12 *PtrDof* genes could be the targets of 15 miRNAs and *PtrDof30* was confirmed as a real target of miRNA. The results help to characterize the stress responses of *PtrDof* genes and promote a better understanding of the construction and function of *Dofs* in *Populus*.

## Supporting Information

S1 Table Primers for qRT-PCR of 39 selected *PtrDof* genes. Primers were designed using Primer Premier 5 (F represents a forward primer; R represents a reverse primer).
(DOC)

S2 Table Primers used for RLM-RACE. (DOC)

S3 Table Motif sequences of *PtrDof* genes identified in *P*. *trichocarpa*. (DOC)

S4 Table Abiotic stress and phytohormone related *cis*-elements. (DOC)

S5 Table Abiotic stress and phytohormone response elements in *PtrDof* gene promoters. (DOC)

S6 Table Details of the Gene Ontology annotation of *PtrDof* sequences. (DOC)

S7 Table The detailed data for gene expression under drought stress of *PtrDofs*. (DOC)

S8 Table The miRNA target predication of *PtrDof* genes. (DOC)
