# Supplementary material for: Characterization of Dof Transcription Factors and Their Responses to Osmotic Stress in Poplar (Populus trichocarpa)
Source: PLoS One. 2017 Jan 17;12(1):e0170210. doi: 10.1371/journal.pone.0170210 (PMC5241002; doi:10.1371/journal.pone.0170210)
Supplement: S1 Table — Primers were designed using Primer Premier 5 (F represents a forward primer; R represents a reverse primer). (DOC) [file pone.0170210.s001.doc]

**S1 Table.** **Primers for qRT-PCR of 39 selected *PtrDof* genes.** Primers were designed using Primer Premier 5 (F represents a forward primer, R represents a reverse primer).

| **Gene name** | **Primer (5'-3')** |
| --- | --- |
| *PtrDof1* | F ATGGCTAGCCAAGAAGAGGG |
|  | R CTTGGCTGGTTAACATTGTAG |
| *PtrDof2* | F GAGGTGGTTGCCGAAAAGGA |
|  | R TGGTCCTCAAAGCAGGACTTAC |
| *PtrDof3* | F AGCCCGATTTTACTGGTGAA |
|  | R TTCTTCAAGTCGTTGCTTCTCT |
| *PtrDof4* | F ACTCTTAGGAGGTGCTGGCT |
|  | GTTTGAAGAATCGCATCGAGGG |
| *PtrDof6* | F ATGATTCAAGAACTCTTAG |
|  | R CATCTCAAGTTCTCTTGAGCT |
| *PtrDof7* | F GTGGTCCTGGTGGGTATTCAG |
|  | R TGAGAGTTTTGACCACCTGAGC |
| *PtrDof8* | F TAACAACCTCAGCGCTCCAC |
|  | R ATGCTGGGAACTGCCAACATT |
| *PtrDof9* | F GTGGCGGAGGCAGGTTATTT |
|  | R TAACGCCGGCAACTCTTACA |
| *PtrDof10* | F GATGTCCCTTGATGGAAATA |
|  | R ATGATCCCAGATTGTTTAGGT |
| *PtrDof11* | F TTGGTGGTGGTTGCAGGAAG |
|  | R CCTGTCGAAAAGTCAGAGATGG |
| *PtrDof12* | F TGTGAAGCCAAATCAAGAGAGG |
|  | R ACCGCCAAGAGTCCAATACC |
| *PtrDof13* | F ATGATTCCTTTGAGAGAAGA |
|  | R GGACATTTAAGAGCTTGCTG |
| *PtrDof14* | F GGAGGGATTGGACCATCAAA |
|  | R AGCTTTAGTGAACCCGGAAG |
| *PtrDof15* | F CAGCTGTGAAAACTGAGGT |
|  | R CCAAGATCCTCTTCCACCAAA |
| *PtrDof16* | F GTGCAAGATGCTTTGTGGTC |
|  | R TGGAAAAACCTCAAACCCCG |
| *PtrDof17* | F CCTGGAATGGAAAATGTTTC |
|  | R TTATGATCAGATACTTCTTT |
| *PtrDof19* | F GTGGGATTTTTTCTGAGATTG |
|  | R AATCAACCCCCCTCCTCCTGTC |
| *PtrDof20* | F TCTCATCATCATCATCATCA |
|  | R TTACTATACCCACTTTCAAG |
| *PtrDof21* | F GAAACAGTGGCACTTTCATG |
|  | R ATTCATCATGCCTGTCCATGA |
| *PtrDof22* | F CTGGCCCAAACTCTCCAACC |
|  | R CTTATCATTCTTAATCCCCA |
| *PtrDof23* | F TGGGTTCTTGGCTCTTAATG |
|  | R CAAACCCATTTTCCTGACCA |
| *PtrDof24* | F CTGCTCAGGAAATGGCTCAC |
|  | R AGATAGTACCTTTTGATGCGGG |
| *PtrDof25* | F TGAAAGATGCCTTTGGATTC |
|  | CAGGATTAGCGTGCAAGACT |
| *PtrDof26* | F CTATTGAGAACCAGGTACAG |
|  | R GTCATTATTGCCATTGTTGC |
| *PtrDof27* | F TTTGGGTTCATCTTTGTCTC |
|  | R GCCATGGTTGAGCGATAGTG |
| *PtrDof28* | F CCTTTTGGGTTCATCTGTGT |
|  | R GCAATGAATGAGTGATGGTG |
| *PtrDof29* | F GTGGATTTGCTTCTAGGGGT |
|  | R AAGCTGTTTCAGCTCACCAAA |
| *PtrDof30* | F GTTGTTGGCATCAAGCGGGC |
|  | R CACCTTGCAAGTAACTCTCGC |
| *PtrDof31* | F TTACACATCAAGTTTTCCTC |
|  | AATATCCTGTAGAATCACCA |
| *PtrDof32* | F AGTGCATCAATTTCCTTTCT |
|  | R GGGCACCCAAATATGGCTTC |
| *PtrDof33* | F TGGCATTCCCAGCTATGCAG |
|  | R TGGCATCGGTGTTGGGATAA |
| *PtrDof34* | F GCTCTCGGAAGATTTGGACA |
|  | R TCCCCGCCAACAAAACCATT |
| *PtrDof35* | F CAAGTGACCTGAATTTTCAA |
|  | R AGATGGTTCAGCACCACCTT |
| *PtrDof36* | F CCTGCTTTGGAGAGGAAGGT |
|  | R CCTCCACCAACAGGAATGCT |
| *PtrDof37* | F TACTTTTCCACTGGGGAAAC |
|  | R TAATAGAACTGGATTTCTCA |
| *PtrDof38* | F TTTGGCGATCAAGTTGTTTGGG |
|  | R ACTCCCTGTGGTTGCCTTCT |
| *PtrDof39* | F GGGTGGAGGGATTGAATCATCA |
|  | R TCACTGGACCAGGAAGATCAG |
| *PtrDof40* | F TTTCTTTGAGTCTCCACCCG |
|  | R TATTTTCTGGGATACCCAAA |
| *PtrDof41* | F GCCATCAACCCAGAACTTTT |
|  | ACATTCCATTCCAATAGCCA |
| *Actin1* | F AGGTTATGCCCTTCCACACG |
|  | R TCGAGGGCAACATACGCAAG |
