# Supplementary material for: Characterization of Dof Transcription Factors and Their Responses to Osmotic Stress in Poplar (Populus trichocarpa)
Source: PLoS One. 2017 Jan 17;12(1):e0170210. doi: 10.1371/journal.pone.0170210 (PMC5241002; doi:10.1371/journal.pone.0170210)
Supplement: S2 Table — (DOC) [file pone.0170210.s002.doc]

**S2 Table. Primers used for RLM-RACE.**

| **Primer Name** | **Primer sequences** |
| --- | --- |
| PtrDof30-RLM-R1 | GTAAATAGCAAGACCAGGCCAC |
| PtrDof30-RLM-R2 | AGACCAGGCCACCCATTATTAC |
