# Supplementary material for: Characterization of Dof Transcription Factors and Their Responses to Osmotic Stress in Poplar (Populus trichocarpa)
Source: PLoS One. 2017 Jan 17;12(1):e0170210. doi: 10.1371/journal.pone.0170210 (PMC5241002; doi:10.1371/journal.pone.0170210)
Supplement: S3 Table — (DOC) [file pone.0170210.s003.doc]

**S3 Table. Motif sequences of *PtrDof* genes identified in *P*. *trichocarpa*.**

| **Motif** | **Width (a.a.)** | **Best possible match** |
| --- | --- | --- |
| 1 | 50 | CPRCDSTNTKFCYYNNYNLTQPRHFCKTCRRYWTKGGALRNVPVGGGCRK |
| 2 | 50 | CIWVPKTLRIDDPSEAAKSSIWATLGIKNDKPDPINGGGLFKAFESKAED |
| 3 | 50 | IMIDEALHQVQAHVMNGVHHPSMKANGTVLTFGSDAPLCDSMASVLNLAD |
| 4 | 19 | MKDPAIKLFGKTIPVHDIQ |
| 5 | 21 | STHVSQLAAVKTHENQDLNLA |
| 6 | 41 | SNSSDEACKGMSKEAVMKDYQGFPPQIPCFPGVPWPYPWNS |
| 7 | 21 | FPMPFYPAPAYWGCTVPGPWN |
| 8 | 41 | PPPPPPPPHVGGSGTAGSIRPGSMADRARLAKIPLPEVALK |
| 9 | 15 | ANPAALERKLRFQED |
| 10 | 21 | EQEDTSIEQEKTLKKPDKIIP |
| 11 | 15 | MVFSSIPVYMDPPDW |
| 12 | 15 | DNTGYWNGMMGGGSW |
| 13 | 47 | DIIGHMPPQPPQLPFMPPLHHLTDFGSGDIGLNFGGIQHQIGATAGG |
| 14 | 14 | GRLLFPFEDLKQQV |
| 15 | 21 | FPLQEFKPTLSFSLDGLGSRY |
