# Supplementary material for: Characterization of Dof Transcription Factors and Their Responses to Osmotic Stress in Poplar (Populus trichocarpa)
Source: PLoS One. 2017 Jan 17;12(1):e0170210. doi: 10.1371/journal.pone.0170210 (PMC5241002; doi:10.1371/journal.pone.0170210)
Supplement: S4 Table — (DOC) [file pone.0170210.s004.doc]

**S4 Table. Abiotic stress and phytohormone related *cis*-elements.**

| **Element** | **Core sequence** | **Function annotation** |
| --- | --- | --- |
| ABRE | TACGGTC | Response to abscisic acid |
| CGTCA-motif | CGTCA | Response to MeJA |
| ERE | ATTTCAAA | Response to ethylene |
| HSE | AAAAAATTTC | Response to heat stress |
| MBS | TAACTG | MYB binding site involved in drought-inducibility |
| TCA-element | GAGAAGAATA | Response to salicylic acid |
| TGACG-motif | TGACG | Response to MeJA |
| W-Box | TTGACC | WRKY binding site involved in abiotic stress responsiveness |
| GARE-motif | TCTGTTG | Response to gibberellin |
| TC-rich repeats | ATTTTCTTCA | Response to defense and stress |
| LTR | CCGAAA | Response to low-temperature |
| P-box | CCTTTTG | Response to gibberellin |
| C-repeat/DRE | TGGCCGAC | Response to cold and dehydration |
