# Supplementary material for: Characterization of Dof Transcription Factors and Their Responses to Osmotic Stress in Poplar (Populus trichocarpa)
Source: PLoS One. 2017 Jan 17;12(1):e0170210. doi: 10.1371/journal.pone.0170210 (PMC5241002; doi:10.1371/journal.pone.0170210)
Supplement: S5 Table — (DOC) [file pone.0170210.s005.doc]

**S5 Table. Abiotic stress and phytohormone response elements in *PtrDof* gene promoters.**

| **Gene name** | ***Cis*-elements related to abiotic stress responsiveness** | | | | | | | |  |  |  |  |
| --- | --- | --- | --- | --- | --- | --- | --- | --- | --- | --- | --- | --- |
|  | **ABRE** | **CGTCA-motif** | **ERE** | **HSE** | **MBS** | **TCA-element** | **TGACG-motif** | **GARE-motif** | **TC-rich repeats** | **LTR** | **P-box** | **C-repeat/DRE** |
| *PtrDof1* |  | ○ | ○ | ○ | ○ | ○ | ○ | ○ |  |  |  |  |
| *PtrDof2* |  | ○ |  |  |  | ○ | ○ | ○ | ○ |  |  |  |
| *PtrDof3* | ○ |  | ○ |  | ○ |  |  |  | ○ |  |  |  |
| *PtrDof4* |  |  | ○ | ○ | ○ |  |  |  | ○ |  |  |  |
| *PtrDof5* |  |  | ○ | ○ |  |  |  |  | ○ | ○ |  |  |
| *PtrDof6* | ○ |  | ○ | ○ | ○ |  |  | ○ | ○ |  |  |  |
| *PtrDof7* |  |  | ○ | ○ |  |  | ○ |  |  | ○ |  |  |
| *PtrDof8* |  | ○ | ○ | ○ |  | ○ | ○ |  | ○ |  | ○ |  |
| *PtrDof9* | ○ |  |  | ○ | ○ | ○ |  |  | ○ |  |  | ○ |
| *PtrDof10* | ○ | ○ |  |  | ○ |  | ○ |  |  | ○ |  |  |
| *PtrDof11* | ○ | ○ |  | ○ | ○ | ○ | ○ | ○ | ○ | ○ | ○ |  |
| *PtrDof12* |  |  | ○ | ○ | ○ | ○ |  | ○ | ○ | ○ | ○ |  |
| *PtrDof13* | ○ |  |  |  |  | ○ |  | ○ | ○ |  |  |  |
| *PtrDof14* | ○ |  | ○ | ○ |  |  |  |  | ○ |  | ○ |  |
| *PtrDof15* |  | ○ |  | ○ |  | ○ | ○ |  | ○ |  | ○ |  |
| *PtrDof16* | ○ | ○ |  | ○ | ○ | ○ | ○ | ○ | ○ |  | ○ |  |
| *PtrDof17* | ○ | ○ |  | ○ |  | ○ | ○ | ○ |  | ○ | ○ |  |
| *PtrDof18* |  |  |  | ○ | ○ |  |  |  | ○ |  |  |  |
| *PtrDof19* | ○ | ○ | ○ | ○ | ○ | ○ | ○ |  | ○ |  |  | ○ |
| *PtrDof20* | ○ |  | ○ | ○ | ○ |  |  |  | ○ |  |  |  |
| *PtrDof21* | ○ | ○ | ○ |  | ○ | ○ | ○ |  | ○ | ○ |  |  |
| *PtrDof22* | ○ |  | ○ | ○ |  | ○ |  |  | ○ |  |  |  |
| *PtrDof23* |  | ○ | ○ | ○ |  |  | ○ |  | ○ | ○ | ○ |  |
| *PtrDof24* |  | ○ | ○ | ○ | ○ | ○ | ○ | ○ | ○ |  |  |  |
| *PtrDof25* | ○ |  |  | ○ | ○ |  |  | ○ | ○ |  |  |  |
| *PtrDof26* | ○ |  |  | ○ | ○ |  |  | ○ | ○ |  |  |  |
| *PtrDof27* | ○ | ○ |  | ○ | ○ |  |  | ○ | ○ |  |  |  |
| *PtrDof28* | ○ | ○ |  | ○ | ○ |  | ○ |  | ○ |  | ○ |  |
| *PtrDof29* | ○ | ○ | ○ | ○ |  | ○ | ○ | ○ | ○ |  |  |  |
| *PtrDof30* |  |  |  | ○ |  | ○ |  | ○ | ○ |  |  |  |
| *PtrDof31* | ○ | ○ |  |  | ○ |  |  |  | ○ |  |  |  |
| *PtrDof32* | ○ |  | ○ | ○ | ○ | ○ |  |  | ○ |  | ○ |  |
| *PtrDof33* |  | ○ |  | ○ | ○ | ○ | ○ | ○ | ○ | ○ |  |  |
| *PtrDof34* | ○ | ○ | ○ | ○ | ○ | ○ |  | ○ |  |  |  |  |
| *PtrDof35* |  | ○ | ○ |  | ○ | ○ | ○ | ○ |  |  |  |  |
| *PtrDof36* | ○ | ○ |  | ○ | ○ | ○ | ○ |  | ○ | ○ | ○ |  |
| *PtrDof37* | ○ | ○ |  |  |  |  | ○ |  | ○ | ○ |  |  |
| *PtrDof38* | ○ |  | ○ | ○ | ○ |  |  | ○ | ○ |  | ○ |  |
| *PtrDof39* | ○ | ○ |  | ○ |  | ○ | ○ |  | ○ |  |  |  |
| *PtrDof40* | ○ |  | ○ |  |  | ○ |  |  | ○ |  |  |  |
| *PtrDof41* |  |  |  |  | ○ | ○ |  |  | ○ |  |  |  |
