# Supplementary material for: Characterization of Dof Transcription Factors and Their Responses to Osmotic Stress in Poplar (Populus trichocarpa)
Source: PLoS One. 2017 Jan 17;12(1):e0170210. doi: 10.1371/journal.pone.0170210 (PMC5241002; doi:10.1371/journal.pone.0170210)
Supplement: S6 Table — (DOC) [file pone.0170210.s006.doc]

**S6 Table. Details of the Gene Ontology annotation of *PtrDof* sequences.**

| **Sequence name** | **GOs** |
| --- | --- |
| *PtrDof1* | F:DNA binding, P:regulation of transcription, DNA-templated |
| *PtrDof2* | F:DNA binding, P:regulation of transcription, DNA-templated |
| *PtrDof3* | F:DNA binding, P:regulation of transcription, DNA-templated |
| *PtrDof4* | F:DNA binding, P:regulation of transcription, DNA-templated |
| *PtrDof5* | F:DNA binding, P:regulation of transcription, DNA-templated |
| *PtrDof6* | F:DNA binding, P:regulation of transcription, DNA-templated |
| *PtrDof7* | F:DNA binding, P:regulation of transcription, DNA-templated |
| *PtrDof8* | C:integral component of membrane, F:DNA binding, P:regulation of transcription, DNA-templated |
| *PtrDof9* | F:DNA binding, P:regulation of transcription, DNA-templated |
| *PtrDof10* | C:integral component of membrane, F:DNA binding, F:transcription factor activity, sequence-specific DNA binding, P:procambium histogenesis, P:phloem or xylem histogenesis, P:positive regulation of transcription, DNA-templated |
| *PtrDof11* | F:DNA binding, P:regulation of transcription, DNA-templated |
| *PtrDof12* | F:DNA binding, P:regulation of transcription, DNA-templated |
| *PtrDof13* | F:DNA binding, F:transcription factor activity, sequence-specific DNA binding, P:regulation of transcription, DNA-templated |
| *PtrDof14* | F:DNA binding, P:regulation of transcription, DNA-templated |
| *PtrDof15* | C:nucleus, F:DNA binding, F:transcription factor activity, sequence-specific DNA binding, P:regulation of transcription, DNA-templated |
| *PtrDof16* | F:DNA binding, P:regulation of transcription, DNA-templated |
| *PtrDof17* | C:nucleus, F:DNA binding, F:transcription factor activity, sequence-specific DNA binding, P:regulation of transcription, DNA-templated |
| *PtrDof18* | F:DNA binding, P:regulation of transcription, DNA-templated |
| *PtrDof19* | F:DNA binding, F:transcription factor activity, sequence-specific DNA binding, P:regulation of transcription, DNA-templated |
| *PtrDof20* | F:DNA binding, P:regulation of transcription, DNA-templated |
| *PtrDof21* | C:integral component of membrane, F:DNA binding, F:transcription factor activity, sequence-specific DNA binding, P:procambium histogenesis, P:phloem or xylem histogenesis, P:positive regulation of transcription, DNA-templated |
| *PtrDof22* | F:DNA binding, P:regulation of transcription, DNA-templated |
| *PtrDof23* | F:DNA binding, P:regulation of transcription, DNA-templated |
| *PtrDof24* | C:integral component of membrane, F:DNA binding, P:regulation of transcription, DNA-templated |
| *PtrDof25* | F:DNA binding, P:regulation of transcription, DNA-templated |
| *PtrDof26* | F:DNA binding, P:regulation of transcription, DNA-templated |
| *PtrDof27* | F:DNA binding, P:regulation of transcription, DNA-templated |
| *PtrDof28* | F:DNA binding, P:regulation of transcription, DNA-templated |
| *PtrDof29* | F:DNA binding, P:regulation of transcription, DNA-templated |
| *PtrDof30* | F:DNA binding, F:transcription factor activity, sequence-specific DNA binding, P:regulation of transcription, DNA-templated, P:cysteine biosynthetic process |
| *PtrDof31* | F:DNA binding, P:regulation of transcription, DNA-templated |
| *PtrDof32* | F:DNA binding, P:regulation of transcription, DNA-templated |
| *PtrDof33* | F:DNA binding, P:regulation of transcription, DNA-templated |
| *PtrDof34* | F:DNA binding, P:regulation of transcription, DNA-templated |
| *PtrDof35* | F:DNA binding, P:regulation of transcription, DNA-templated |
| *PtrDof36* | F:DNA binding, P:regulation of transcription, DNA-templated |
| *PtrDof37* | F:DNA binding, P:regulation of transcription, DNA-templated |
| *PtrDof38* | F:DNA binding, P:regulation of transcription, DNA-templated |
| *PtrDof39* | F:DNA binding, P:regulation of transcription, DNA-templated |
| *PtrDof40* | F:DNA binding, P:regulation of transcription, DNA-templated |
| *PtrDof41* | F:DNA binding, P:regulation of transcription, DNA-templated |
| P, F and C represent biosynthetic process, molecular function and cellular component, respectively. | |
