# Supplementary material for: Characterization of Dof Transcription Factors and Their Responses to Osmotic Stress in Poplar (Populus trichocarpa)
Source: PLoS One. 2017 Jan 17;12(1):e0170210. doi: 10.1371/journal.pone.0170210 (PMC5241002; doi:10.1371/journal.pone.0170210)
Supplement: S7 Table — (DOC) [file pone.0170210.s007.doc]

**S7 Table. The detailed data for gene expression under drought stress of *PtrDofs***.

| **Gene** | **Leaves-control** | **Leaves-drought** | **Leaves-drought-2** | **Roots-control** | **Roots-drought** |
| --- | --- | --- | --- | --- | --- |
| *PtrDof1* | 2.86 | 2.51 | 3.16 | 4 | 3.4 |
| *PtrDof2* | 4.58 | 4.3 | 4.22 | 6.63 | 6.23 |
| *PtrDof3* | 1.75 | 2.12 | 3.82 |  |  |
| *PtrDof4* | 3.57 | 4.37 | 3.38 | 5.53 | 5.59 |
| *PtrDof5* |  |  |  |  |  |
| *PtrDof6* | 5.43 | 6.05 | 4.55 | 7.98 | 8.03 |
| *PtrDof7* | 3.02 | 1.73 | 5.94 | 3.86 | 3.4 |
| *PtrDof8* | 2.58 | 2.88 | 1.84 | 3.24 | 2.72 |
| *PtrDof9* | 8.14 | 8.91 | 8.27 | 7.26 | 7.66 |
| *PtrDof10* | 5.33 | 5.99 | 4.55 | 5.67 | 4.77 |
| *PtrDof11* |  |  |  |  |  |
| *PtrDof12* | 3.35 | 3.62 | 4.38 | 5.66 | 5.7 |
| *PtrDof13* | 6.56 | 6.99 | 6.68 | 6.8 | 6.45 |
| *PtrDof14* | 2.58 | 2.42 | 2.96 | 2.86 | 3.17 |
| *PtrDof15* | 5.26 | 6.64 | 6.78 | 7.49 | 8.5 |
| *PtrDof16* | 5.2 | 5.97 | 5.14 | 2.53 | 3.22 |
| *PtrDof17* | 2.09 | 3.54 | 4.57 | 5.72 | 5.41 |
| *PtrDof18* |  |  |  |  |  |
| *PtrDof19* | 6.04 | 6.52 | 5.55 | 6.02 | 7.08 |
| *PtrDof20* | 4.04 | 4.44 | 4.28 | 7.82 | 7.47 |
| *PtrDof21* | 5.12 | 5.98 | 4.13 | 5.5 | 4.87 |
| *PtrDof22* | 6.89 | 5.95 | 5.88 | 5.61 | 5.6 |
| *PtrDof23* | 2.09 | 3 | 2.74 | 7.94 | 7.9 |
| *PtrDof24* | 5.21 | 3.69 | 5.2 | 7.05 | 6.6 |
| *PtrDof25* | 7.64 | 8.48 | 9.18 | 6.03 | 7.41 |
| *PtrDof26* | 3.8 | 3.83 | 3.82 | 3.24 | 3.27 |
| *PtrDof27* | 3.89 | 4.96 | 5.51 | 7.47 | 7.7 |
| *PtrDof28* | 6.42 | 7.17 | 9.2 | 5.88 | 6.24 |
| *PtrDof29* | 4.45 | 4.39 | 4.4 | 9.24 | 8.61 |
| *PtrDof30* | 5.08 | 4.73 | 5.86 | 5.62 | 6.12 |
| *PtrDof31* | 5.75 | 5.95 | 6.24 | 6.99 | 6.95 |
| *PtrDof32* | 4.99 | 6 | 5.2 | 6.67 | 6.24 |
| *PtrDof33* | 6.73 | 6.94 | 6.48 | 9.83 | 9.72 |
| *PtrDof34* | 1.27 | 3.62 | 3.7 | 7.8 | 7.19 |
| *PtrDof35* | 3.52 | 3.58 | 2.82 | 4.38 | 4.39 |
| *PtrDof36* | 5.43 | 5.69 | 5.77 | 6.97 | 6.75 |
| *PtrDof37* | 6.91 | 7.1 | 7.41 | 5.34 | 6.44 |
| *PtrDof38* | 5.58 | 5.5 | 5.02 | 3.93 | 3.59 |
| *PtrDof39* | 5.36 | 6.6 | 5.95 | 7.16 | 7.27 |
| *PtrDof40* | 4.36 | 4.91 | 5.1 | 5.29 | 4.86 |
| *PtrDof41* | 4.15 | 4.76 | 3.9 | 7.85 | 7.32 |
