# Supplementary material for: Characterization of Dof Transcription Factors and Their Responses to Osmotic Stress in Poplar (Populus trichocarpa)
Source: PLoS One. 2017 Jan 17;12(1):e0170210. doi: 10.1371/journal.pone.0170210 (PMC5241002; doi:10.1371/journal.pone.0170210)
Supplement: S8 Table — (DOC) [file pone.0170210.s008.doc]

**S8 Table. The miRNA target predication of *PtrDof* genes.**

| **miRNA** | **Target** | **Expectation** | **UPE** | **miRNA_start** | **miRNA_end** | **Target_start** | **Target_end** | **miRNA_aligned_fragment** | **Target_aligned_fragment** | **Inhibition** |
| --- | --- | --- | --- | --- | --- | --- | --- | --- | --- | --- |
| ptc-miR472b | *PtrDof30* | 3 | 23.812 | 1 | 21 | 1170 | 1190 | CCUACCCACCUCAACCCUUUU | GGAUGGGUGGGUUUGGGGAGG | Translation |
| ptc-miRf10002-akr | *PtrDof8* | 2.5 | 21.259 | 1 | 21 | 2982 | 3001 | ACCCGAAGGGAAUGUUAGACG | UGGGCUUCCCUUCCAAUUUGC | Translation |
| ptc-miRf10003-akr | *PtrDof20* | 3 | 13.941 | 1 | 22 | 1371 | 1392 | AGGUACACCCGAAGGGAAUGUU | UUCAACUGGGUUUCCUUUACAA | Cleavage |
| ptc-miRf10053-akr | *PtrDof13* | 2.5 | 17.023 | 1 | 21 | 398 | 418 | GUCGUCGUCGUUGAUGAUGUC | CAGCAGCAGCAGCAGCUGCAG | Cleavage |
| ptc-miRf10426-akr | *PtrDof35* | 2 | 20.252 | 1 | 21 | 1254 | 1274 | ACCCAUACCACCACCUCGGUU | UGGUUAUGGUGGUGGAGCUGA | Cleavage |
| ptc-miRf10476-akr | *PtrDof25* | 3 | 10.526 | 1 | 20 | 793 | 812 | GUGGUACUUUUUGGUUGAAA | UAUCAUUGAAAACCAAUUUU | Cleavage |
| ptc-miRf10540-akr | *PtrDof40* | 3 | 17.169 | 1 | 21 | 1171 | 1191 | GCUAAGUCUUUAGUCGUCGUU | CGGUUCAGGAAUGAGUAGCAG | Translation |
| ptc-miRf10677-akr | *PtrDof24* | 3 | 18.652 | 1 | 20 | 4028 | 4047 | AAGAGAAACGAACGAUGCCC | UUCUUUUAGCUUGCUAGGGG | Cleavage |
| ptc-miRf10897-akr | *PtrDof1* | 3 | 20.826 | 1 | 24 | 297 | 321 | GUCACCAUUCUCGAACC-CUGGUUC | UAGAUGUAAGAGCAUGGAGACCAAG | Cleavage |
| ptc-miRf10957-akr | *PtrDof13* | 2.5 | 17.023 | 1 | 22 | 397 | 418 | UGUCGUCGUCGUUGAUGAUGUC | ACAGCAGCAGCAGCAGCUGCAG | Cleavage |
| ptc-miRf11023-akr | *PtrDof12* | 3 | 19.445 | 1 | 20 | 579 | 598 | ACACUUAGAACUACUACGAG | UGUCAAUCUUGGUGGUGUUU | Cleavage |
| ptc-miRf11148-akr | *PtrDof40* | 3 | 17.169 | 1 | 21 | 1171 | 1191 | GCUAAGUCUUUAAUCGUCGUU | CGGUUCAGGAAUGAGUAGCAG | Translation |
| ptc-miRf11606-akr | *PtrDof32* | 3 | 17.646 | 1 | 20 | 1433 | 1452 | CUCUAGGGUAGACUGCUAGG | GAGAACCUAUCUGAUGAUCA | Cleavage |
| ptc-miRf12020-akr | *PtrDof12* | 3 | 19.445 | 1 | 20 | 579 | 598 | ACACUUAGAACUACUACGAG | UGUCAAUCUUGGUGGUGUUU | Cleavage |
| ptc-miRf12124-akr | *PtrDof28* | 3 | 21.534 | 1 | 22 | 1358 | 1379 | CUUUUGAUGUAGCGACGGCUAG | GAGAAUUACGUCUCUGCUGAUC | Translation |
